# Withdrawal of antiepileptic drugs in patients with low grade and anaplastic glioma after long-term seizure freedom: a prospective observational study

**DOI:** 10.1007/s11060-019-03117-y

**Published:** 2019-02-18

**Authors:** M. Kerkhof, J. A. F. Koekkoek, M. J. Vos, M. J. van den Bent, W. Taal, T. J. Postma, J. E. C. Bromberg, M. C. M. Kouwenhoven, L. Dirven, J. C. Reijneveld, M. J. B. Taphoorn

**Affiliations:** 1 Department of Neurology, Haaglanden Medical Center, PO Box 2191, 2501 VC The Hague, The Netherlands; 2 Department of Neurology, Leiden University Medical Center, Leiden, The Netherlands; 3 Brain Tumor Center at Erasmus MC Cancer Institute, Rotterdam, The Netherlands; 4 Brain Tumor Center Amsterdam at VU University Medical Center, Amsterdam, The Netherlands

**Keywords:** Glioma, Epilepsy, Withdrawal, Antiepileptic drugs

## Abstract

**Background:**

When glioma patients experience long-term seizure freedom the question arises whether antiepileptic drugs (AEDs) should be continued. As no prospective studies exist on seizure recurrence in glioma patients after AED withdrawal, we evaluated the decision-making process to withdraw AEDs in glioma patients, and seizure outcome after withdrawal.

**Methods:**

Patients with a histologically confirmed low grade or anaplastic glioma were included. Eligible patients were seizure free ≥ 1 year from the date of last antitumor treatment, or ≥ 2 years since the last seizure when seizures occurred after the end of the last antitumor treatment. Patients and neuro-oncologists made a shared decision on the preferred AED treatment (i.e. AED withdrawal or continuation). Primary outcomes were: (1) outcome of the shared decision-making process and (2) rate of seizure recurrence.

**Results:**

Eighty-three patients fulfilled all eligibility criteria. However, in 12/83 (14%) patients, the neuro-oncologist had serious objections to AED withdrawal. Therefore, 71/83 (86%) patients were analyzed; In 46/71 (65%) patients it was decided to withdraw AED treatment. In the withdrawal group, 26% (12/46) had seizure recurrence during follow-up. Seven of these 12 patients (58%) had tumor progression, of which three within 3 months after seizure recurrence. In the AED continuation group, 8% (2/25) of patients had seizure recurrence of which one had tumor progression.

**Conclusion:**

In 65% of patients a shared decision was made to withdraw AEDs, of which 26% had seizure recurrence. AED withdrawal should only be considered in carefully selected patients with a presumed low risk of tumor progression.

## Introduction

Low grade gliomas are a group of primary brain tumors supposedly developing from supportive tissue cells, such as oligodendrocytes and astrocytes, or neural stem cells. In the presence of microvascular proliferation and necrosis, these tumors are designated as anaplastic gliomas. A fundamental shift in the diagnosis of these tumors is effectuated by the increasing importance of molecular markers in the histopathology of these tumors [1, 2].

Most patients with low grade glioma develop seizures during the course of their disease. In general, patients with low grade gliomas [World Health Organization (WHO) grade II] appear to have a much higher seizure incidence (up to 60–90%) compared to patients with anaplastic gliomas (WHO grade III, 40–60%) [3–6]. Epilepsy in patients with glioma may be difficult to treat as 15–50% of patients do not become seizure free despite extensive treatment with antiepileptic drugs (AEDs) [3, 4]. Epilepsy in patients with brain tumors is characterized by localization-related seizures, manifesting as focal seizures either with or without focal to bilateral tonic-clonic seizures. In clinical practice, there is no doubt that glioma patients who develop seizures require treatment with AEDs. To achieve adequate seizure control, levetiracetam and valproic acid are the mostly supported treatment options [5], but alternative AEDs as lamotrigine, lacosamide, topiramate, zonisamide or pregabaline also have shown a favorable efficacy and toxicity profile and limited interactions with other drugs such as chemotherapeutic agents [6–9]. Still, in 20–40% of glioma patients AED side-effects occur, such as somnolence, dizziness, fatigue, cognitive disturbances, and mood or behavioral changes [5, 10]. Besides seizures, the tumor itself and antitumor treatments, the cumulative effects of AED treatment are also likely to contribute to cognitive dysfunction, behavioral changes and a decrease in quality of life [10–13]. The potential benefits and harms should therefore be weighted when choosing to start a specific AED.

Evidence exists that antitumor treatment for glioma also contributes to a reduction in seizure frequency; after surgical resection or radiotherapy, respectively 53–87% and 32–75% of patients with low grade glioma become seizure free [14]. Also chemotherapy treatment results in a ≥ 50% reduction in seizure frequency in 48–78% of patients [13, 15–19]. Consequently, tumor-directed treatments are increasingly recognized as potentially effective options leading to seizure control [20].

In the light of potential side effects and costs of long-term AED use and the efficacy of anti-tumor treatment regarding seizures, the question arises whether withdrawal of AEDs after an interval of seizure freedom should be considered [5]. Based on retrospective studies, a seizure recurrence rate after withdrawal between 12.5 and 27% has been reported in patients with mostly intra-axial brain tumors [5]. Currently, it is unknown if glioma patients and their physicians are willing to withdraw AEDs after long-term seizure freedom, and more importantly, no prospective studies exist on the risk of seizure recurrence in glioma patients after AED withdrawal. Therefore, we studied both the decision-making process of glioma patients and their neuro-oncologists to withdraw or continue AEDs after long-term seizure freedom, as well as the rate of seizure recurrences.

## Methods

### Design

A prospective, observational study was conducted. Details on the study design can be found in the published study protocol [21].

### Participants

Participants were recruited from January 2014 until May 2016 from the outpatient clinic in three large tertiary referral centers for brain tumor patients in the Netherlands: Haaglanden Medical Center The Hague, Brain Tumor Center Amsterdam at VU University Medical Center Amsterdam and Brain Tumor Center at the Erasmus MC Cancer Institute Rotterdam. Consecutive patients visiting the outpatient clinic were screened for eligibility based on information in their medical charts. The inclusion criteria were as follows; (1) adults > 18 years of age, (2) histologically confirmed WHO grade II–III glioma, (3) history of epilepsy defined as at least one seizure, except for acute provoked seizures, for which treated with AEDs, (4) clinically and radiologically stable disease for at least 12 months, and (5) seizure freedom for at least 12 months from the date of last surgery, irradiation or chemotherapy cycle, or seizure freedom for at least 24 months from the last seizure when a seizure occurred after the last antitumor treatment. As no formal definition of long-term seizure freedom exists in literature, the current definition (at least > 12 months) was based on expert opinion. In case patients fulfilled the inclusion criteria, first the treating neuro-oncologist had to decide if it was safe to withdraw AEDs. If not, the reason for exclusion was registered. In patients in whom it was considered to be safe to withdraw AEDs, the neuro-oncologist and patient needed to make a shared decision on either continuation or withdrawal of AEDs. Patients had to give informed consent prior to inclusion in the study. The medical ethical committees of all participating centers approved the study.

### Withdrawal or continuation of AEDs

Patients were included in the withdrawal group in case it was decided to withdraw AEDs, or in the continuation group in case of any objection to withdraw AEDs. The reason for AED continuation was registered separately.

In the withdrawal group, each AED was tapered according to a fixed schedule; a step-wise 50% dose reduction every 2 weeks. In case of using more than one AED, the latest added AED was withdrawn first. In the continuation group, no changes were made in antiepileptic therapy. All participants were evaluated at 3, 6, 12 and 18 months. During these follow-up assessments, data were collected about changes in AED treatment, seizure recurrence, type and date of seizures, and the date of tumor progression. In case of seizure recurrence, dosages of AEDs were adapted or AEDs were (re)started according to the expertise of the treating neuro-oncologist. The primary outcomes were the decision-making process of AED withdrawal, and the rate of seizure recurrence. Secondary outcomes were type of epilepsy at seizure recurrence, time between inclusion in study and seizure recurrence, and the association between seizure recurrence and tumor progression, WHO grade, time of seizure freedom before inclusion, and time since diagnosis.

### Statistical analysis

Baseline patient characteristics and information about seizure and tumor recurrence were reported using descriptive statistics. Differences between groups were assessed with the Chi-Squared test (χ^2^) or Fisher’s Exact test in case of categorical variables. For continuous variables the independent T-test or Mann–Whitney U test were used, depending on the distribution of the variable. Statistical analyses were performed using SPSS version 23 (SPSS, Chicago, IL). All tests were exploratory, two-tailed, and *p* < 0.05 was considered to be statistically significant.

## Results

### Willingness to withdraw AEDs

**Fig. 1:**
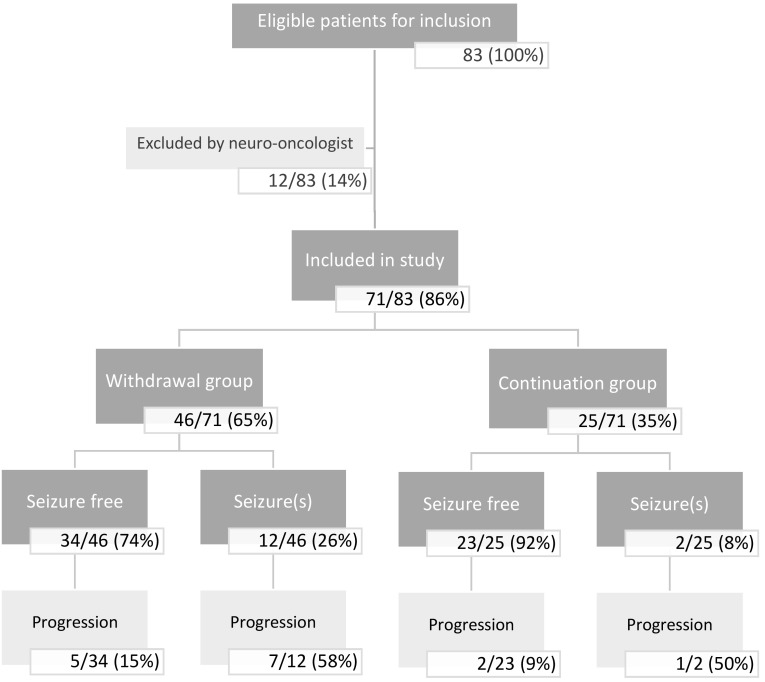
Flowchart patients. Eligibility, AED group and seizure recurrence

A total of 83 patients fulfilled all eligibility criteria. Of these, 71 (86%) were included in the study (Fig. 1). In 12 patients (14%) the neuro-oncologist had serious objections to AED withdrawal. The reported reasons for exclusion were: a presumed high risk of recurrent seizures due to history of refractory seizures (n = 3), severe cognitive dysfunction (n = 2), psychologically not stable enough for withdrawal (n = 6), and another medical indication for AED use (n = 1). Of the 71 patients approved for inclusion by the treating neuro-oncologist, a shared decision to withdraw AED(s) was made in 46 patients (65%) and to continue AED(s) in 25 patients (35%). The most frequently reported reasons to continue AEDs reported by patients were the possibility to lose their driving license in case of a new seizure (n = 8), and fear for recurrent seizures (n = 8). Four patients reported both the consequences for the driving license as well as fear as reason to continue AED treatment, while five patients did not report any reason.

### Patient and tumor characteristics

The baseline characteristics of the 71 included patients are shown in Table 1. Patients in the withdrawal and continuation group were similar with respect to all clinical and sociodemographic variables. The mean age in the withdrawal group was 50 (range 24–72) years compared to 53 (range 28–79) years in the continuation group (p = 0.24). The withdrawal group and continuation group consisted of 24/46 (52%) and 17/25 (68%) WHO grade II tumors, and 22/46 (48%) and 8/25 (32%) WHO grade III tumors (p = 0.20), respectively. In the withdrawal group 18/46 (39%) tumors had loss of 1p/19q versus 11/25 (44%) tumors in the continuation group (p = 0.40). In 20 patients, 15 in the withdrawal and 5 in the continuation group, 1p/19q status was unknown. Before inclusion in the study, 33/71 (46%) patients had focal to bilateral tonic-clonic seizures, 13/71 (18%) had focal seizures, 11/71 (15%) both focal to bilateral tonic-clonic and focal seizures, and for the remaining patients seizure type was unknown (n = 14, 20%). Eleven patients (11/46, 24%) in the withdrawal group had at least once tumor progression compared to 3 (3/25, 12%) patients in the continuation group (p = 0.12). Most patients used levetiracetam or valproic acid as AED (58% vs. 23%), with no differences between groups (p = 0.57).

**Table 1:** Patient and tumor characteristics

Patient and tumor characteristics	AED withdrawal group (n = 46) (%)	AED continuation group (n = 25) (%)	Sign (p-value)
Age (mean years, range)	50 (24–72)	53 (28–79)	0.24
Hospital			< 0.005
VUmc	18 (39)	4 (16)	
HMC	18 (39)	4 (16)	
EMC	10 (22)	17 (68)	
Diagnosed (mean years, range)	7.2 (3.1–19.5)	7.9 (3.8–15.7)	0.12
WHO grade glioma			0.20
Grade II	24 (52)	17 (68)	
Grade III	22 (48)	8 (32)	
LOH 1p/19q			0.40
1p/19q codeleted	18 (39)	11 (44)	
1p/19q not codeleted	13 (28)	9 (36)	
Unknown	15 (33)	5 (20)	
Type epilepsy			0.59
Focal to bilateral tonic clonic	21 (46)	12 (48)	
Focal	10 (22)	3 (12)	
Combination	9 (20)	2 (8)	
Unknown	6 (13)	8 (32)	
Duration seizure free before inclusion (median years, range)	2.9 (1–12.8)	4.1 (1–20)	0.06
Tumor progression before inclusion			0.12
No progression	35 (76)	22 (88)	
≥ One progression	11 (24)	3 (12)	
Latest anti-tumor treatment			0.25
Surgery	6 (13)	2 (8)	
Radiotherapy	26 (57)	11 (44)	
Chemotherapy	14 (30)	13 (52)	
AED use			0.57
VPA	10 (22)	6 (24)	
LEV	27 (59)	14 (56)	
LAM	2 (4)	1 (4)	
PHT	3 (7)	0 (0)	
CBZ	3 (7)	4 (16)	
LAC	1 (2)	0 (0)	
Type therapy			0.24
Monotherapy	40 (87)	19 (76)	
Polytherapy (> 1 AED)	6 (13)	6 (24)	

*AED* antiepileptic drug, *VPA* valproic acid, *LEV* levetiracetam, *LAM* lamotrigine, *PHT* phenytoin, *CBZ* carbamazepine, *LAC* lacosamide

### Follow-up withdrawal group

The median follow-up in the withdrawal group was 2.2 (range 0.8–3.8) years (Table 2). At the end of follow-up, 12/46 (26%) patients who withdrew AEDs had seizure recurrence (Fig. 1). Of the 12 patients with seizure recurrence, 8 (67%) patients had a focal seizure, two patients (17%) had a focal to bilateral tonic-clonic seizure, one patient (8%) had a status epilepticus consisting of a focal seizure with impaired awareness, and one patient (8%) probably had a nocturnal seizure (Table 3). Seven out of 12 patients (58%) had seizure recurrence within 3 months after the start of withdrawal. In all 12 patients, AED treatment was restarted according to the expertise of the treating neuro-oncologist. Two of these patients had repeated seizures after restarting AED treatment; one patient had one focal seizure while the other patient had frequent focal seizures, even after higher dosages of AEDs. The patient with the status epilepticus was admitted to the hospital for 1 day. Another patient with seizure recurrence during AED withdrawal tapered the AEDs faster than advised.

**Table 2:** Seizure recurrence in relation to tumor progression

	Withdrawal group (N = 46)	Continuation group (N = 25)	p-value
Median duration follow-up (years)	2.2 (range 0.8–3.8)	1.7 (range 0.8–2.9)	0.03
Seizure recurrence	12 (26%)	2 (8%)	0.67
Tumor progression	12 (26%)^a^	3 (12%)^b^	0.12

^a^7/12 with seizure recurrence had tumor progression

^b^1/2 with seizure recurrence had tumor progression

**Table 3:** Patients with seizure recurrence and/or tumor progression in both study groups

Type tumor	1p/19q	Group	Type seizure	Inclusion-seizure (mo)	Seizure-progression (mo)	Inclusion-progression (mo)
Seizure recurrence and progression						
OD II	Codeleted	Withdrawal	Focal	2.5	0	2.5
A III	Intact	Withdrawal	Focal	13	1	14
OA II	Unknown	Withdrawal	Focal	5.5	18	23.5
A II	Intact	Withdrawal	Focal	4.5	0	4.5
A II	Intact	Withdrawal	Focal	2	18	20
A II	Unknown	Withdrawal	Focal to bilateral tonic-clonic	4	8.5	12.5
A II	Intact	Withdrawal	Focal	1.5	19	20.5
OD III	Codeleted	Continue	Focal	6	4	10
Seizure recurrence without progression						
A III	Intact	Withdrawal	Focal	30	–	–
OD III	Codeleted	Withdrawal	Nocturnal	3	–	–
OA III	Intact	Withdrawal	Focal	2	–	–
OD II	Unknown	Withdrawal	Status epilepticus	10	–	–
OA II	Unknown	Withdrawal	Focal to bilateral tonic-clonic	21	–	–
OD II	Intact	Continue	Focal	18	–	–
Progression without seizure recurrence						
OD III	Intact	Withdrawal	–	–	–	31
OD II	Unknown	Withdrawal	–	–	–	20
OD III	Unknown	Withdrawal	–	–	–	7
OD II	Codeleted	Continue	–	–	–	11
OD III	Codeleted	Withdrawal	–	–	–	26
A II	Intact	Continue	–	–	–	17
A II	Intact	Withdrawal	–	–	–	12

*OD* oligodendroglioma, *A* astrocytoma, *OA* oligo-astrocytoma, *II* WHO grade II, *III* WHO grade III, *mo* months

There were no significant differences in WHO grade, time of seizure freedom before inclusion, or time since diagnosis in the group with seizure recurrence compared to the group without seizure recurrence (Table 3). The 1p/19q status of patients without seizure recurrence differed significantly from the patients with seizure recurrence; 44% (15/34) were 1p/19q co-deleted in the group without seizure recurrence versus 25% (3/12) in the group with seizure recurrence (p = 0.04).

Twenty-six percent (12/46) of patients in the withdrawal group showed tumor progression during the follow-up period. This included 7/12 patients (58%) with seizure recurrence. Of these, three patients had tumor progression within 3 months after seizure recurrence. In the other four patients the interval between tumor progression and seizure recurrence was more than 3 months (range 4–19) (Table 3). Progression occurred significantly more often in patients with seizure recurrence (7/12, 58%) than in patients without seizure recurrence (5/34, 15%, p = 0.006).

### Follow-up continuation group

The median follow-up was 1.7 (range 0.8–2.9) years in the continuation group. In this group, 12% (3/25) showed tumor progression. Two out of 25 (8%) patients in this group had seizure recurrence, which was a focal seizure in both cases. One of the two patients with seizure recurrence had tumor progression 4 months later (Table 3).

## Discussion

In this study, the decision-making process of patients and doctors to withdraw antiepileptic drugs in clinically and radiologically stable low grade and anaplastic glioma patients that had long-term seizure freedom (> 1 year) was studied, as well as the rate of seizure recurrence after AED withdrawal. This is the first study in which the recurrence rate of seizures in glioma patients is evaluated prospectively. In low grade as well as anaplastic glioma patients with longstanding stable disease, neuro-oncologists often question whether continuation of AED use is necessary to remain seizure free after years of seizure freedom. Positive outcome of drug withdrawal may include improvement of cognitive functioning and abolishment of (subtle) side-effects of AEDs such as tiredness, which is especially important in this socially active patient population [22]. Although not all patients were deemed eligible for inclusion by neuro-oncologists, we showed that the majority of the eligible patients (65%) were willing to withdraw AED treatment after long-term seizure freedom.

After a mean follow-up of 2.2 years, the recurrence rate of seizures after AED withdrawal was 26% (12/46). The risk of seizure relapse after AED withdrawal in glioma patients appears to be comparable with the general epilepsy population with non-brain tumor related epilepsy [23–25]. In the general epilepsy population, followed for variable periods of time ranging from 3 months to 23 years, a recurrence rate of 12–66% was reported [5, 25, 26]. Predictors for seizure recurrence after withdrawal in the general epilepsy population include AED polytherapy, longer duration of active epilepsy, having experienced seizures after the start of AED treatment, and having an abnormal EEG [26]. EEG testing was not performed in this study, as this is not common in brain tumor-related epilepsy. In this patient population, the results of EEG testing typically do not change the decision to alter or withdraw AEDs [27].

From this study no definite conclusions can be drawn whether AED withdrawal after long-term seizure freedom in glioma patients is advisable as the seizure recurrence rate is still considerable. When making a shared-decision on possible withdrawal of AEDs, the potential positive effects of AED withdrawal should be weighed against the risk of seizure recurrence. Both neuro-oncologists and patients are, in varying degrees, cautious in withdrawing AEDs due to fear for renewed seizures and the potential consequences such as seizure-related injuries. The psychosocial impact of recurrent seizures is also large; seizures can be embarrassing, obstruct professional careers, make patients more dependent on others, and lead to a temporary loss of a driving license [26]. Indeed, the fear for seizure recurrence and the possible loss of their driving license were the two most important reasons for patients to continue AED treatment in our study. The data presented in this prospective study can be used to better inform patients and neuro-oncologists about the risk of seizure recurrence, helping to make well-considered decisions.

In all but one patient, AEDs were withdrawn in line with the study protocol. In this single patient, who experienced seizure recurrence, AEDs were tapered faster than advised. In theory, this quick tapering might have contributed to the recurrence of seizures, warranting caution in the way AEDs are withdrawn in glioma patients. Unfortunately, one patient in our study was admitted to the hospital with a focal status epilepticus. It is noteworthy that this patient fulfilled the eligibility criteria for inclusion. However, it appeared that this patient had a medical history of status epilepticus (twice). Based on this finding, it could be argued not to withdraw AEDs in seizure free patients with a history of status epilepticus.

It is of interest that more than half of the patients with seizure recurrence (7/12, 58%) in the withdrawal group had tumor progression during study follow-up. Those seven patients were a median of 6.5 years (range 3.4–13) ago diagnosed with a glioma and two of them had already tumor progression prior to inclusion in the study. Indeed, three of the seven patients had tumor progression within 3 months after seizure recurrence. In these patients, seizure recurrence might have been an indication for progression of the tumor as there is some evidence available that seizures are a surrogate marker for tumor progression [28–30]. Previously, seizure recurrence or worse seizure control was found to be associated with tumor progression following first-line treatment [31]. Furthermore, the risk of tumor progression in low grade glioma patients is four times higher in case of seizure recurrence [32]. Considerably more patients had tumor progression in the withdrawal group compared to the continuation group. This finding may have influenced the risk of seizure recurrence in the withdrawal group. It is also possible that the study groups were not well-balanced with respect to risk of progression, although no significant differences were found in the baseline patient- and tumor-related characteristics. Moreover, it might be that the higher amount of tumor progression in the withdrawal group is caused by the discontinuation of AED itself, as conflicting evidence exists that valproic acid might have an anti-tumor effect as well. Several retrospective studies in patients with glioblastoma have suggested that valproic acid moderately improves survival in glioma patients treated with temozolomide, although a larger meta-analysis could not confirm this [8, 33–35]. Interestingly, within the withdrawal group, the subgroup with seizure recurrence had significantly more often tumor progression and the prognostic more unfavorable intact 1p/19q status. Although based on small numbers, seizure recurrence after withdrawal seems to be associated with the absence of 1p/19q codeletion, and is also related to a higher risk of tumor progression within this cohort. In this study, the risk of seizure recurrence after withdrawal was not associated with WHO grade, time after diagnosis, or duration of seizure freedom.

Given the inclusion criteria for our study, with stability of disease for at least a year as a major criterium, we purposely left out glioblastoma patients who have a limited prognosis. For the non-glioblastoma patients, we think that both grade II and grade III patients are of interest, since both low-grade and anaplastic patients with a relatively long survival might specifically benefit from withdrawal of antiepileptic drugs.

Due to the small numbers, a multivariable analysis to assess which factors were independently associated with seizure recurrence could not be performed.

Due to ethical objections to randomize patients with regard to AED withdrawal, a prospective observational study design was chosen, including both the decision-making process and an evaluation of seizure outcome. For the decision to withdraw AEDs, no specific decision-making model was used. Instead, the process depended on the preferred communication method of the neuro-oncologists [36, 37]. Although both study groups seem to be well-balanced in both patient- and tumor-related characteristics, a risk exists for confounding-by-indication due to the non-randomized study design, which might have influenced the results.

Although not systematically assessed, we did receive positive responses from patients about the withdrawal of their medication. Patients subjectively reported a better concentration or mood. Nevertheless, it would have been interesting to evaluate the impact of seizure recurrence on patients’ wellbeing, and to ask patients whether being medication-free outweighs experiencing a new seizure.

## Conclusion

Neuro-oncologists and glioma patients are now better informed about the risk of seizure recurrence after AED withdrawal. The results presented here can be used in shared decision-making during consultations. Our advice would be to withdraw AEDs only in carefully selected patients. The possible negative side effects of AEDs, the effect of antitumor treatment on seizure frequency, and patients’ requests to withdraw medication suggest that an attempt to withdraw AEDs can be considered in patients with low grade or anaplastic glioma experiencing long-term seizure freedom.
